# Physical Activity Time, Alcohol Consumption, Mediterranean Diet, and Anxiety in Education Science Students

**DOI:** 10.3390/ejihpe14010006

**Published:** 2023-12-25

**Authors:** Daniel Sanz-Martín, Félix Zurita-Ortega, Eduardo Melguizo-Ibáñez, José Manuel Alonso-Vargas, Rafael Caracuel-Cáliz, José Luis Ubago-Jiménez

**Affiliations:** 1 Faculty of Humanities and Social Sciences, Universidad Isabel I, 09003 Burgos, Spain; daniel.sanz6718@ui1.es; 2 Department of Didactics Musical, Plastic and Corporal Expression, Faculty of Education Science, University of Granada, 18071 Granada, Spain; felixzo@ugr.es (F.Z.-O.); josemalonsov@correo.ugr.es (J.M.A.-V.); jlubago@ugr.es (J.L.U.-J.); 3 Faculty of Education Science, Universidad Internacional de Valencia (VIU), 46002 Valencia, Spain; rafaelfrancisco.caracuelcaliz@unir.net

**Keywords:** healthy lifestyle, active lifestyle, negative emotions, university education

## Abstract

Student lifestyles change during university. This research aimed to classify university students according to their levels of physical activity, alcohol consumption, adherence to the Mediterranean diet, and anxiety and studied the relationships between the variables using a multigroup equation model according to gender. The sample was composed of 549 participants (M = 23.06; S.D. = 6.22), of whom 409 were women and 140 were men. Validated and adapted instruments such as the Beck Anxiety Inventory, the PREDIMED Questionnaire, and the Alcohol Use Disorder Identification Test were used. The data revealed four clusters through Ward’s method and the k-means method. Regarding the exploratory model, differences were found in the effects of the variables according to sex. In conclusion, alcohol consumption was positively associated with the Mediterranean diet, and physical activity was negatively associated with the Mediterranean diet and anxiety.

## 1. Introduction

The teenage years are currently considered a key period in human development [1]. This fact is due to a large number of physical, psychological, and emotional changes that occur [2]. This stage of human growth is highly important [1,2]. Several studies show problems related to healthy lifestyle acquisition in this group [3]. Gender differences in choosing a healthy lifestyle have been observed [1]. Women tend to be more sedentary than men with respect to following an active lifestyle [4]. They are also more likely to show greater adherence to a healthy dietary pattern [1]. The transition to university is also a sensitive time, as young people begin to make their own nutritional choices [5].

A progressive disengagement from healthy dietary patterns can be seen among the university population [6]. Undergraduates show relatively low adherence to the Mediterranean diet [7]. Recent research indicates that positive adherence to this dietary pattern brings health benefits such as improved blood pressure; a reduced prevalence of metabolic syndrome; and improvements in high-density lipoprotein cholesterol, triacylglycerol, and glucose concentrations [8,9]. The Mediterranean dietary pattern is characterized by the consumption of foods such as fruits, vegetables, olive oil, legumes, oily fish, and dairy products [8]. In addition, the Mediterranean diet is characterized by low levels of consumption of processed red meat and saturated fats as well as small amounts of alcoholic beverages [8].

The consumption of alcoholic beverages plays a key role in university life [10]. Binge drinking large amounts of alcoholic beverages in short periods of time after a period of abstinence has been observed [11]. This phenomenon has been found to be highly prevalent among university students [11]. It has an adverse impact on the health of young people, increasing the risk of brain damage and cancer [12,13]. Evidence shows that many university students engage in this type of behavior in the presence of negative emotional states [14], of which anxiety predominates [15].

University students are faced with situations that lead to increased levels of anxiety [15]. It has been observed that in order to cope with anxiety symptoms, alcohol is consumed, resulting in its presence in the bloodstream [16]. Drinking behavior at the university stage may be due to different reasons and situations [17]. Students with a high level of anxious symptomatology may be vulnerable to drinking [7]. Anxiety has been positively associated with drinking alcohol to cope with specific social situations [18,19,20]. Identifying the different situations that lead to increased anxiety and subsequent alcohol consumption could facilitate the prevention of alcohol misuse [19,20].

The relationship between alcohol consumption and health status is very complex [21,22]. A study by Leasure et al. [21] found that the profile of the moderate drinker includes positive adherence to a healthy diet as well as regular physical activity. Physically active people demonstrate moderate consumption of alcoholic beverages [23]. There is a positive relationship between physical activity and alcohol consumption [4]. It has been shown that alcohol consumption is linked to participation in victories, which encourages alcohol consumption [24]. Habitual alcohol consumption has been shown to have a negative effect on muscle function [25]. Regular alcohol consumption leads to the depletion of amino acids and glucose in muscles [25]. This leads to an inadequate energy supply and the impairment of metabolic processes during sport [25]. In view of this, physical activity has been shown to reduce the oxidative damage exerted by ethanol on the liver and myocardium [25].

Research similar to the present study exists [1,5,7,10,15]. The study presented herein provides a very comprehensive overview of the current state of the variables and the problems addressed in the literature. A cluster analysis was carried out to classify university students according to their physical and sporting health and their psychosocial variables. In order to make the research more rigorous, a multigroup explanatory model was developed to study differences in the effects according to the sex of the participants. This type of analysis offers a very complete vision of the current state of the contextualized variables and problems addressed in this research.

Considering the above, the aims of this study were (a) to classify and identify existing differences in university students according to their levels of physical activity, alcohol consumption, adherence to the Mediterranean diet, and anxiety and (b) to study the relationships between alcohol consumption, Mediterranean diet adherence, weekly physical activity time, and anxiety through a multigroup equation model according to gender.

## 2. Materials and Methods

### 2.1. Participants and Study Design

This study adopted an ex post facto (non-experimental) exploratory, cross-sectional, descriptive correlational design. The research presented here was conducted on 549 students from the University of Granada. Looking at the characteristics of the sample, the ages of the participants were between 18 and 24 years (23.06 ± 6.22). The study sample consisted of 409 girls (74.5%) and 140 boys (25.5%). There was only one inclusion criterion: being an education science student. Each young student who met the criterion was invited to participate. In addition, the young participants were assured of their anonymity and guaranteed that the data would be processed exclusively for scientific purposes. In order to reach as many students as possible, this study was advertised on the department’s website and on the researchers’ social media. This allowed a larger number of participants to be reached.

### 2.2. Instruments

The instruments used in this research have been validated. The main characteristics of these instruments are described below:

**Sociodemographic information and intensity of physical activities:** These variables included the sex (male/female) and age of the participants. To measure weekly physical activity time, the criteria established by the World Health Organization were followed [26]. In this case, this question was categorized into three responses (less than 150 min, between 150 and 300 min, and more than 300 min) [27,28]. 

**Beck Anxiety Inventory** [29]**:** Due to the characteristics of this research, the version by Sanz and Navarro [30] was used. This version of the original questionnaire consists of 21 items. Each of these questions is assessed using a four-dimensional Likert scale. Regarding the reliability of the instrument, a Cronbach’s alpha value of α = 0.963 was obtained.

**Alcohol Use Disorders Identification Test (AUDIT)** [31]: Considering the characteristics of the sample, the version by García-Carretero [32] was used. The instrument consists of 10 items assessed on a Likert scale (0 = never; 4 = every day). For this study, a degree of reliability of α = 0.801 was found. Based on the final score, alcohol consumption was classified into the following levels: Low-Risk Consumption, Risky Consumption, and High Consumption.

**PREDIMED Questionnaire** [33]**:** The Spanish version was used for this research [34]. This questionnaire consists of 14 items that can be answered positively or negatively. Depending on the final score, the response was categorized into three levels (Low Adherence, Medium Adherence, and High Adherence).

### 2.3. Procedure and Ethical Aspects 

The ethical criteria set out in the Declaration of Helsinki were followed during data collection. Before being given access to the study, students were asked for their informed consent to participate in the research. Furthermore, they were assured that the data would be treated anonymously and used for scientific purposes. Finally, to ensure that the research complied with research ethics principles, the research was supervised by an ethics committee of the University of Granada (2966/CEIH/2022). 

### 2.4. Analysis of the Data

To address the first research objective (to classify university students according to their levels of physical activity, alcohol consumption, Mediterranean diet, and anxiety), IBM SPPS 26.0 software (International Business Machines Corporation, Armonk, NY, USA) was used. The analysis had three distinct parts: the initial part involved the descriptive correlational statistics of the whole sample, the second part involved clustering, and the final part involved the corroboration of the classification and descriptive correlational statistics of each cluster.

In the first phase, the data were cleaned, and the descriptive correlational statistical analysis of the study variables was carried out. In addition, the data of the variables were typified (z-scores) to allow a cluster analysis to be performed [35]. Subsequently, nine outliers (greater than 3.0 or less than −3.0) were removed so as not to distort the results of the following tests [36]. After eliminating outliers, the final sample consisted of 549 participants. The normality of the variables was analyzed using the Kolmogorov–Smirnov test, asymmetries, and kurtosis. A non-normal distribution was obtained. The mean values of the variables were compared according to the sex of the participants using the (two-sample) Mann–Whitney U test. In addition, correlations were analyzed using Spearman’s test.

The cluster analysis makes it possible to exhaustively detail the levels of the research variables and their correlations according to the most homogeneous groupings of people possible. In this way, health promotion proposals designed in the future to improve the levels obtained in this study will be more precise for each group of participants identified, focusing for example on the most deficient or priority factors. This type of intervention design means that proposals will be more efficient than if they were designed for a single and more heterogeneous group of participants (for example, in terms of financial resources or intervention time). 

In the second phase, Spanish university students were classified according to their levels of physical activity, alcohol consumption, Mediterranean diet, and anxiety according to the process established by Hair et al. [36]. First, Ward’s agglomerative hierarchical procedure based on the squared Euclidean distance was used to determine the optimal number of clusters. Ward’s method minimizes the distance between subjects in each cluster and avoids the emergence of long chains [37]. Next, the k-means method was used to classify the cases into the groups established in the previous process. The joint application of Ward’s method and the k-means method is common in social research [35].

In the third phase of the statistical analysis, a MANOVA test was used to validate the cluster classification of the previous phase. The independent variable was cluster membership, and the dependent variables were physical activity, alcohol consumption, Mediterranean diet, and anxiety. The result of the Pillai trace test identified the effect between variables [38] and that of Levene’s test for the equality of error variances. Subsequently, post hoc Tukey (Levene’s *p*-value ≤ 0.05) or Games–Howell (Levene’s *p*-value > 0.05) data were selected. Finally, a descriptive correlational analysis of the variables in each of the clusters was carried out according to the sex of the participants. The Kolmogorov–Smirnov, (two-sample) Mann–Whitney U, Chi-square, and Cramer’s V tests were used.

**Figure 1:**
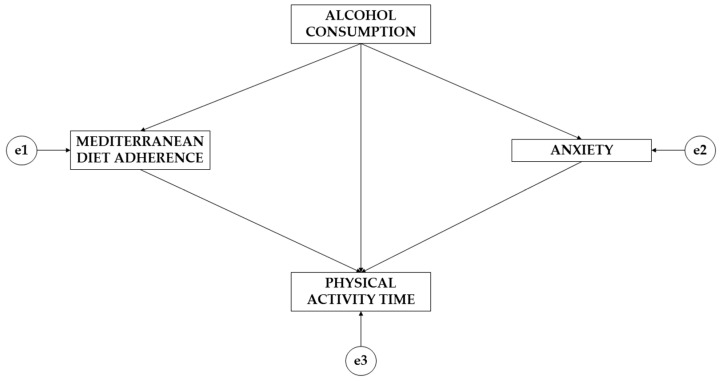
Theoretical model developed for this research.

To address the second research objective (to study the relationships between alcohol consumption, Mediterranean diet adherence, weekly physical activity time, and anxiety through a multigroup equation model according to gender), IBM SPSS Amos 26.0 software (IBM Corp., Armonk, NY, USA) was used to develop the exploratory analysis. The theoretical model (Figure 1) consisted of four variables. Regarding the types of variables, three acted as endogenous variables (adherence to the Mediterranean diet; anxiety; and physical activity time) and one acted as an exogenous variable (alcohol consumption). For the endogenous variables, a causal explanation was obtained. This was performed based on the observed associations between the indicators and the degree of reliability of the measurement process, which allowed the inclusion of error in the model itself. The arrows were unidirectional, allowing the effect to be studied in only one direction. For the study of statistically significant differences, two levels of significance were established (*p* ≤ 0.05; *p* ≤ 0.001).

To assess the model fit, the criteria established by Maydeu-Olivares [39] and Kyriazos [40] were followed. Initially, the fit of the Chi-square test was assessed, with non-significant values showing a good fit. Then, specific adjustments (goodness of fit index, comparative fit index, and incremental reliability index) needed to be considered, with values above 0.900 denoting a good fit. The value of the root-mean-square approximation also needed to be considered, which needed to be less than 0.100. Likewise, following the criteria established by Tenembaum and Eklund [41], the size and susceptibility of the sample needed to be considered, so the Tucker–Lewis index was included, which needed to be greater than or equal to 0.900.

Based on the pathways that the model offers (Figure 1), a brief description is given below. Note that the model is made up of four variables, with one acting as an exogenous variable (alcohol consumption) and three acting as endogenous variables (Mediterranean diet adherence, anxiety, and physical activity time). It is observed that alcohol consumption acts on adherence to the Mediterranean diet, anxiety, and physical activity. In turn, the variables adherence to the Mediterranean diet and anxiety exert a unidirectional effect on physical activity time.

## 3. Results

### 3.1. Cluster Analysis 

Spanish university students generally practice more than three hours of physical activity per week, do not consume alcohol, obtain a mean score of 0.18 ± 0.11 for Mediterranean diet consumption, and obtain a mean score of 0.77 ± 0.60 for anxious symptomatology. Table 1 shows the descriptive statistics according to the sex of the participants.

In addition, there are significant differences in the practice of physical activity (*x*^2^ (2) = 16.27, *p* ≤ 0.001; *V* = 0.17), alcohol consumption (*x*^2^ (2) = 6.90, *p* = 0.03; *V* = 0.11), and anxious symptomatology (U = 21616.50, *p* ≤ 0.001) regarding gender. 

Table 2 presents the mean descriptive statistics (z-scores) of the variables and their correlations.

Based on the results of the squared Euclidean distance proximity matrix, the clustering history, and the dendrogram obtained from the use of Ward’s method, it was decided to choose four clusters. Table 3 presents the descriptive statistics of the four clusters according to the classification of the k-means method. Cluster 1 participants are characterized by the highest average anxiety score (1.38 ± 0.66). Cluster 2 is the largest cluster (38.62%) and is characterized by the lowest averages for alcohol consumption (−0.80 ± 0.00) and anxiety (−0.59 ± 0.43). Students in cluster 3 have the lowest levels of physical activity (−0.99 ± 0.62) and the highest levels of Mediterranean diet adherence (0.64 ± 0.83). Participants in cluster 4 have the highest averages for physical activity (0.69 ± 0.54) and alcohol consumption (1.18 ± 0.54) and the lowest level of Mediterranean diet adherence (−0.31 ± 0.89).

The values of the four ranking variables for each of the four clusters do not follow normal distributions, according to the Kolmogorov–Smirnov test (*p* ≤ 0.05). Furthermore, the skewness ranges from −0.82 to 3.18, and the kurtosis ranges from −1.98 to 8.29. 

The MANOVA test validated the classification of the four clusters, as the Pillai trace values were *F* (12.1632) = 192.99; *p* ≤ 0.001; *f* = 1.76. 

The results of the Levene’s test for the Mediterranean diet were non-significant (F (3.545) = 1.93; *p* = 0.12); Tukey’s post hoc test was therefore applied. In contrast, the results for physical activity time (F (3.545) = 49.77), alcohol consumption (F (3.545) = 36.63), and anxious symptomatology (F (3.545) = 18.31) were significant (*p* ≤ 0.001), so the Games–Howell post hoc test was used. 

Table 4 shows the post hoc multiple comparisons between the four clusters according to the four study variables.

Table 5 shows the descriptive statistics for each research variable according to the sex of the participants and the cluster to which they belong. 

In cluster 1, there are significant differences in alcohol consumption (*x*^2^ (2) = 10.13, *p* = 0.001; *V* = 0.32) and anxiety (U = 1074.50, *p* = 0.03) regarding gender. In cluster 2, significant differences were found in the practice of physical activity (*x*^2^ (2) = 8.53, *p* = 0.01; *V* = 0.20) and anxiety (U = 3285, *p* = 0.02) regarding sex. In cluster 3, there are only significant differences in the practice of physical activity (*x*^2^ (2) = 12.59, *p* < 0.001; *V* = 0.33). Finally, in cluster 4, there are significant differences in anxiety (U = 983.50, *p* < 0.001).

Table 6 presents the descriptive statistics and Spearman’s correlations of the variables for each cluster. In the alcohol consumption variable of the second cluster, the standard deviation is not positive (equal to zero), so the normality of the variable, the skewness, the kurtosis, and the correlations cannot be calculated. The significant correlations are mostly positive and weak (0.10 < *r* < 0.30), except for the relationship between alcohol consumption and the Mediterranean diet, which is negative in the third cluster; the relationship between the Mediterranean diet and anxiety, which is negative in the fourth cluster; and the relationship between physical activity and the Mediterranean diet, which is positive and moderate (*r* = 0.33, *p* ≤ 0.01) in the third cluster. 

### 3.2. Structural Equation Model Results

In the structural equation model suggested for males, a good fit was found for the different fit indices. The Chi-square test showed a non-significant value (Chi-square = 4.011; degrees of freedom = 2; probability level = 0.135). The values obtained for the comparative fit index, normalized fit index, incremental fit index, and Tucker–Lewis index were 0.939, 0.988, 0.967, and 0.956, respectively. The mean square error of the approximation analysis had a value of 0.059.

The proposed structural equation model for females obtained a good fit for different fit indices. The Chi-square test showed a non-significant value (Chi-square = 1.067; degrees of freedom = 1; probability level = 0.302). The values obtained for the comparative fit index, normalized fit index, incremental fit index, and Tucker–Lewis index were 0.910, 0.994, 0.939, and 0.964, respectively. The mean square error of the approximation analysis was 0.013.

**Figure 2:**
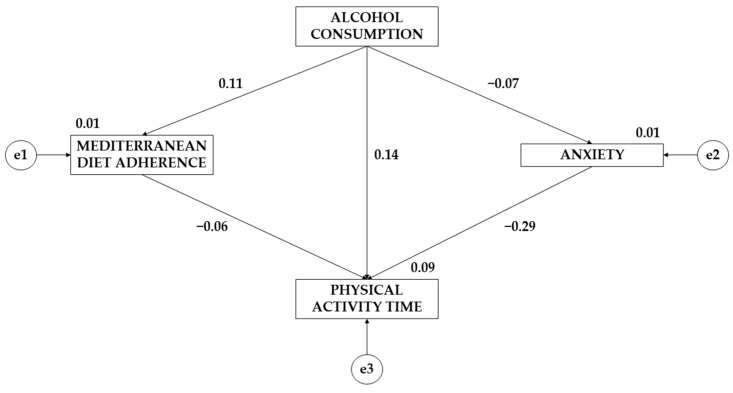
Standardized regression weights together with the theoretical model for the male gender.

**Figure 3:**
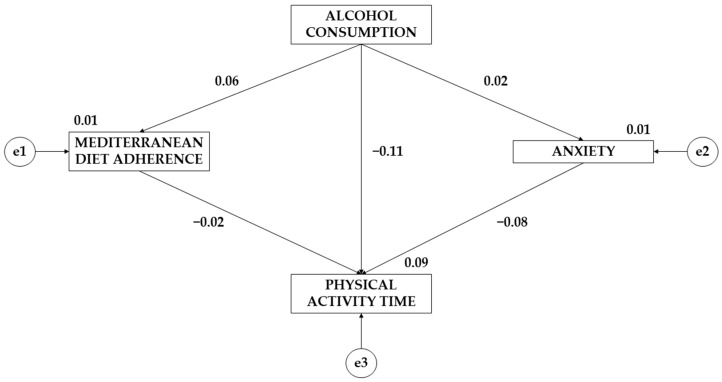
Standardized regression weights together with the theoretical model for the female population.

Table 7 and Figure 2, as well as Table 8 and Figure 3, show the standardized regression weights of the equation models. Concerning differences between alcohol consumption and adherence to the Mediterranean diet, males show a larger effect than females (β = 0.109; β = 0.061). Regarding alcohol consumption’s effect on anxiety, males show a negative effect (β = −0.073) while females show a positive effect (β = 0.017). For the effect of adherence to the Mediterranean diet on weekly physical activity time, a negative effect is observed for both sexes, which is greater for males (β = −0.061; β = −0.024). The effect of anxiety on weekly physical activity time is negative for both sexes but is greater for males (β = −0.287; β = −0.024). Finally, a positive effect of alcohol consumption on physical activity time is observed for males (β = 0.144), but a negative effect is observed for females (β = −0.115).

## 4. Discussion

This study aimed to classify university students according to their physical activity levels, alcohol consumption, Mediterranean diet, and anxiety and to study the relationships between alcohol consumption, adherence to the Mediterranean diet, weekly physical activity time, and anxiety using a multigroup equation model according to gender. In line with these aims, the following discussion will compare these results with findings from previous studies. Based on physical activity levels, alcohol consumption, adherence to the Mediterranean diet, and anxiety levels, four clusters were established. This clustering was also validated using a MANOVA test. Significant differences were found between the mean values of each variable in one cluster compared to other clusters, except for the Mediterranean diet adherence between students in the second and fourth groups. Students from the first cluster have higher mean values for anxiety. Moreover, these levels are positively and significantly correlated with alcohol consumption. Students in the second group have the lowest levels of alcohol consumption and anxiety. There is also a positive and significant correlation between physical activity and adherence to the Mediterranean diet. In the third group, the participants showed the lowest mean values for physical activity and the highest values for Mediterranean diet adherence. Also, there is a positive and significant correlation between physical activity and alcohol consumption and a negative correlation between alcohol consumption and Mediterranean diet adherence. Finally, the fourth group of students is characterized by the highest physical activity and alcohol consumption levels and the lowest levels of diet adherence. In this group, a positive and significant relationship was found between physical activity and alcohol consumption, and a negative relationship was found between physical activity and alcohol consumption.

Furthermore, positive correlations were found in this study between the Mediterranean diet adherence and anxiety levels in each of the identified groups. Zurita-Ortega et al. [42] also found a negative relationship between adherence to diet and mental health problems such as anxiety. Meanwhile, Devonport et al. [43] showed that anxiety can also lead to increased food consumption. This could indicate that study participants, in the presence of anxiety symptoms, may have a similar increase in food intake but not in quality of diet. In addition, negative associations have been shown with the risks of other mental health problems [44,45,46]. Regarding the association between alcohol consumption and anxiety levels, this study showed that the mean levels are low-risk and their relationships are positive, and this is also true in all four clusters. This type of relationship was also found in the study by Jaén-Cortés et al. [47], but there it was significant. Seitz and Mueller [48] reported that heavy alcohol consumption was related to a lower anxiety level. Based on the findings of these studies, it may be the case that the students did not have high anxiety levels during the period when the questionnaires were administered, as they were in a period of lower academic or examination demands [49].

Concerning weekly physical activity time, 77.41% of the university students (85.51% of males and 74.70% of females) met the physical activity recommendations for their age group (at least 150 min of moderate–vigorous physical activity per week) [26]. These sex differences in physical activity levels are consistent with other studies [50,51,52]. The present study showed a positive association in all four groups, with a significant association in groups 2 and 4. Similar results were obtained by Bennasar-Veny et al. [53] and Tárra-ga-López et al. [54]. 

A negative and significant relationship between the participants’ mean physical activity levels and anxiety was also shown in this study. The relationships among the other variables are not significant. However, a well-fitted multigroup equation model was obtained to show the direct and indirect relationships between the study variables for the whole sample, which was valid for both genders. According to this model, alcohol consumption influences physical activity time both directly and indirectly through its influence on Mediterranean diet adherence and anxiety. In addition, there is a positive relationship between alcohol consumption and diet adherence, a negative relationship between diet adherence and physical activity time, and a negative relationship between anxiety levels and physical activity time. The relationship between alcohol consumption and physical activity is positive in the male model and negative in the female model. Similarly, the relationship between alcohol consumption and anxiety is negative in the male model and positive in the female model. Therefore, it can be said that there is still a need to understand the healthy habits of each specific population group and their conditioning factors [55]. Moreover, it is necessary to be prudent in the analysis of the determinants of lifestyle habits because mistakes can be made by analyzing only two factors directly related to each other. However, another factor not being considered may be indirectly influencing this relationship [56]. Similarly, interventions targeting multiple lifestyle habits and aimed at promoting health among university students would have a greater effect when designed jointly compared to designing them for each habit separately [53]. Additionally, in light of the widely established positive association between health improvement and human capital [57,58], promoting healthier lifestyles among university students would also have a positive effect on the acquisition of human capital, which is a key factor in modern economic growth [59,60].

This study has several limitations. Due to the cross-sectional design of the study, the obtained findings refer to a specific point in time and may have differed over time. It should be taken into account that the sample belongs to a very specific and concrete geographical area. Moreover, most of the sample is female. Another limitation is the instrument used to collect physical activity, as it was a questionnaire. Although there are other more accurate instruments to measure physical activity, this one was used since it allows information to be obtained from a large group of people in a reasonable time [57]. It is widely used in epidemiological studies [58], and it is based on the WHO’s practical recommendations [26].

Based on the obtained research findings, it is possible to identify a few research lines. It would be helpful to perform similar studies with other groups, for example, to compare the results according to another variable that could be a determining factor: age. In addition, a longitudinal study could be conducted to identify how healthy habits differ and the relationships between them. It would be interesting to analyze how other health determinants influence those already analyzed and investigated. In addition, it would be convenient to design health promotion programs adapted to the different profiles of the clusters, as this would increase their effectiveness. It would be interesting to add new study variables for future research. One of them would be the motivational climate since this is how the motives for engaging in physical activity are oriented. This research offers a high level of application in the area of sport science. The first application concerns psychological factors. Many athletes experience significant bouts of anxiety. If this is not properly treated, it can lead to a lack of control of the athletes’ diets. Another aspect would be related to the positive pursuit of a healthy lifestyle. Nutrition plays a key role in achieving high sporting performance. Linkages between anxiety and task performance have been observed. When expected results are not achieved, episodes of anxiety are experienced. These anxiety levels are often reduced by the intake of alcoholic beverages and high-calorie foods.

Furthermore, some limitations exist in this research. The first limitation is related to the sample, as more than two thirds are women. Even though a significant error was obtained from the sample under study, this sample does not support generalizations at the national or international levels. It should be highlighted that the included students belong to education-science-related degrees. However, this research provides evidence about how correlations between the study variables differ according to heterogeneous groups or more homogeneous groups of participants. Further international research should take this into account. The instruments used, although validated by the scientific community, have an intrinsic measurement error. The sample is also homogeneous, with about three quarters of the participants being female. Indeed, the students belonging to the educational sciences area, where females have higher participation than males, explains the high percentage of females. It should be noted that the results should be interpreted with moderation. The students belong to a specific study field (educational sciences) and to a very specific geographical area (Southern Spain). No generalizations can be made to samples from other geographical areas or to other fields of study.

## 5. Conclusions

Undergraduate students can be categorized into four clusters according to their physical activity levels, alcohol consumption, Mediterranean diet, and anxiety. In the first cluster, participants with higher anxiety levels are included. In the second cluster, participants with lower anxiety levels are included. In the third cluster, participants with lower physical activity levels are included, while in the fourth cluster, participants with higher physical activity and alcohol consumption levels and lower levels of Mediterranean diet adherence are included.

Designing a health promotion proposal for cluster 1 participants that focuses on increasing physical activity levels and reducing anxiety levels would be appropriate. In addition, reducing anxiety levels would indirectly reduce alcohol consumption levels. Suggestions designed for students in cluster 2 should focus on improving Mediterranean diet adherence. In addition, they would indirectly improve physical activity levels. For students in cluster 3, suggestions to improve the Mediterranean diet could be emphasized, although these levels are the best of the four clusters. The aim would be indirect, whereby through diet, the levels of alcohol consumption and anxiety would be reduced. Afterwards, it would be useful to improve the levels of physical activity. Finally, interventions to improve dietary adherence should be prioritized for participants in group 4.

In the theoretical model, goodness-of-fit was found, which allowed the identification of associations between alcohol consumption, Mediterranean diet adherence, weekly physical activity time, and anxiety independent of the participants’ sex. For these models, alcohol consumption is positively associated with the Mediterranean diet. Likewise, physical activity is negatively associated with the Mediterranean diet and anxiety. There are also significant differences between physical activity, alcohol consumption, and anxiety according to gender.

## Figures and Tables

**Table 1:** Descriptive statistics of the research variables according to gender.

Variable		N or Mean	Sex (N or Mean)	
			Male	Female
Physical Activity Time	Less than 150 min	124	20	104
	Between 150 and 300 min	167	33	134
	More than 300 min	258	85	173
Alcohol Consumption	Low-Risk Consumption	320	69	251
	Risky Consumption	213	62	151
	High Consumption	16	7	9
Mediterranean Diet	Mean score	0.18 ± 0.11	0.18 ± 0.11	0.18 ± 0.12
Anxiety	Mean score	0.77 ± 0.60	0.64 ± 0.65	0.81 ± 0.58

**Table 2:** Descriptive statistics and z-score correlations of the research variables.

	M (SD)	CI (95%)	Kolmogorov–Smirnov	Skewness	Kurtosis	2	3	4
**1. Physical activity**	0.01 (0.99)	−0.01/0.09	0.30	−0.48	−1.28	−0.04	−0.06	−0.16 **
**2. Alcohol consumption**	0.01 (0.99)	−0.08/0.09	0.37	0.73	−0.53	-	0.07	0.01
**3. Mediterranean diet**	0.10 (0.98)	−0.08/0.09	0.11	−0.07	0.01	-	-	0.06
**4. Anxiety**	−0.04 (0.95)	−0.12/0.04	0.14	0.09	0.05	-	-	-

**Note:** ** Correlation is significant at the 0.01 level (bilateral).

**Table 3:** Descriptive statistics of the four k-means clusters.

	N (%)	Sex (N)		Cluster Center (z-Cores)			
		Male	Female	Physical Activity	Alcohol Intake	Mediterranean Diet	Anxiety
**Cluster 1**	102 (18.58)	20	82	−0.01 (1.04)	−0.66 (0.49)	0.21 (1.01)	1.38 (0.66)
**Cluster 2**	212 (38.62)	53	159	0.16 (0.93)	−0.80 (0.00)	−0.25 (0.92)	−0.59 (0.43)
**Cluster 3**	113 (20.58)	20	93	−0.99 (0.62)	0.84 (0.70)	0.64 (0.83)	0.02 (0.81)
**Cluster 4**	122 (22.22)	45	77	0.69 (0.54)	1.18 (0.54)	−0.31 (0.89)	−0.31 (0.70)

**Table 4:** Post hoc multiple comparisons of variables in the clusters.

	Cluster (I)	Cluster (J)	Difference of Means (I–J)	S.D.	Sig.	Confidence Interval (95%)	
						Lower Limit	Upper Limit
**PA**	1	2	−0.18	0.12	0.46	−0.49	0.14
	1	3	0.98	0.12	*	0.67	1.29
	1	4	−0.70	0.11	*	−1.00	−0.41
	2	3	1.16	0.09	*	0.93	1.38
	2	4	−0.52	0.08	*	−0.73	−0.32
	3	4	−1.68	0.08	*	−1.88	−1.48
**AC**	1	2	0.14	0.05	0.02	0.02	0.27
	1	3	−1.50	0.08	*	−1.71	−1.29
	1	4	−1.84	0.07	*	−2.01	−1.66
	2	3	−1.64	0.07	*	−1.81	−1.47
	2	4	−1.98	0.05	*	−2.11	−1.85
	3	4	−0.34	0.08	*	−0.55	−0.12
**MD**	1	2	0.47	0.11	*	0.18	0.75
	1	3	−0.42	0.12	0.01	−0.75	−0.10
	1	4	0.52	0.12	*	0.21	0.84
	2	3	−0.89	0.11	*	−1.16	−0.61
	2	4	0.06	0.10	0.95	−0.21	0.32
	3	4	0.95	0.12	*	0.64	1.25
**AN**	1	2	1.97	0.07	*	1.78	2.16
	1	3	1.36	0.10	*	1.10	1.62
	1	4	1.69	0.09	*	1.45	1.92
	2	3	−0.61	0.08	*	−0.82	−0.40
	2	4	−0.28	0.07	*	−0.46	−0.10
	3	4	0.33	0.10	0.01	0.07	0.58

**Note:** physical activity (PA); alcohol consumption (AC); Mediterranean diet (MD); anxiety (AN); * *p* ≤ 0.001.

**Table 5:** Descriptive statistics for each cluster according to the sex of the participants.

Cluster	Variable		% or Mean	Sex (N or Mean)	
				Male	Female
**Cluster 1**	**Physical Activity Time**	Less than 150 min	25.49	25	25.61
		Between 150 and 300 min	26.47	20	28.05
		More than 300 min	48.04	55	46.34
	**Alcohol Consumption**	Low-Risk Consumption	92.16	75	96.34
		Risky Consumption	0	0	0
		High Consumption	7.84	25	3.66
	**Mediterranean diet**	Mean score	0.21 ± 0.12	0.21 ± 0.13	0.21 ± 0.12
	**Anxiety**	Mean score	1.67 ± 0.42	1.87 ± 0.49	1.62 ± 0.39
**Cluster 2**	**Physical Activity Time**	Less than 150 min	16.04	20.75	14.47
		Between 150 and 300 min	31.13	15.10	36.48
		More than 300 min	52.83	64.15	49.05
	**Alcohol Consumption**	Low-Risk Consumption	100	100	100
		Risky Consumption	0	0	0
		High Consumption	0	0	0
	**Mediterranean diet**	Mean score	0.15 ± 0.11	0.16 ± 0.10	0.15 ± 0.11
	**Anxiety**	Mean score	0.42 ± 0.27	0.34 ± 0.26	0.44 ± 0.27
**Cluster 3**	**Physical Activity Time**	Less than 150 min	55.75	20	63.44
		Between 150 and 300 min	44.25	80	36.56
		More than 300 min	0	0	0
	**Alcohol Consumption**	Low-Risk Consumption	12.38	5	13.97
		Risky Consumption	84.08	90	82.80
		High Consumption	3.54	5	3.23
	**Mediterranean diet**	Mean score	0.26 ± 0.10	0.28 ± 0.06	0.25 ± 0.10
	**Anxiety**	Mean score	0.80 ± 0.51	0.74 ± 0.57	0.82 ± 0.50
**Cluster 4**	**Physical Activity Time**	Less than 150 min	0.82	0	1.30
		Between 150 and 300 min	19.67	11.11	24.68
		More than 300 min	79.51	88.89	74.02
	**Alcohol Consumption**	Low-Risk Consumption	0	0	0
		Risky Consumption	90.16	86.67	92.21
		High Consumption	9.84	13.33	7.79
	**Mediterranean diet**	Mean score	0.15 ± 0.11	0.15 ± 0.11	0.14 ± 0.10
	**Anxiety**	Mean score	0.60 ± 0.44	0.40 ± 0.35	0.71 ± 0.45

**Table 6:** Descriptive statistics and correlations between variables in each cluster.

		Kolmogorov–Smirnov	Skewness	Kurtosis	2	3	4
Cluster 1	PA	0.31 ***	−0.45	−1.41	0.05	0.07	−0.05
	AC	0.54 ***	3.18	8.29	-	−0.03	0.35 **
	MD	0.12 ***	0.09	−0.24	-	-	−0.05
	AN	0.09 *	0.28	−0.74	-	-	-
Cluster 2	PA	0.33 ***	−0.72	−0.86	NA	0.20 **	−0.12
	AC	NA	NA	NA	-	NA	NA
	MD	0.15 ***	−0.25	−0.09	-	-	−0.09
	AN	0.11 ***	0.50	−0.49	-	-	-
Cluster 3	PA	0.37 ***	0.24	−1.98	0.21 *	0.14	−0.06
	AC	0.47 ***	−0.82	3.08	-	−0.19 *	0.15
	MD	0.16 ***	−0.01	−0.20	-	-	−0.01
	AN	0.12 ***	0.71	0.15	-	-	-
Cluster 4	PA	0.49 ***	−1.73	1.86	−0.17	0.33 **	−0.11
	AC	0.53 ***	2.73	5.55	-	0.15	0.05
	MD	0.16 ***	−0.11	0.45	-	-	−0.28 **
	AN	0.12 ***	0.72	−0.37	-	-	-

**Note:** physical activity (PA); alcohol consumption (AC); Mediterranean diet (MD); anxiety (AN); not applicable (NA); *p* ≤ 0.001 (***); *p* ≤ 0.01 (**); *p* ≤ 0.05 (*).

**Table 7:** Standardized regression weights for male gender.

Associations between Variables	R.W.				S.R.W.
	Estimations	S.E.	C.R.	*p*	Estimations
MD ← AC	0.021	0.016	1.296	0.195	0.109
AN ← AC	−0.085	0.098	−0.865	0.387	−0.073
PA ← MD	−0.402	0.527	−0.764	0.445	−0.061
PA ← AN	−0.313	0.088	−3.573	≤0.001	−0.287
PA ← AC	0.183	0.102	1.788	0.074	0.144

**Note:** physical activity (PA); alcohol consumption (AC); Mediterranean diet (MD); anxiety (AN).

**Table 8:** Standardized regression weights for female gender.

Associations between Variables	R.W.				S.R.W.
	Estimations	S.E.	C.R.	*p*	Estimations
MD ← AC	0.013	0.011	1.237	0.216	0.061
AN ← AC	0.020	0.056	0.355	0.722	0.017
PA ← MD	−0.159	0.327	−0.487	0.626	−0.024
PA ← AN	−0.111	0.064	1.739	0.082	−0.084
PA ← AC	−0.171	0.072	−2.361	≤0.05	−0.115

## Data Availability

The data used to support the findings of the current study are available from the corresponding author upon request.
